# Critical role of the mTOR pathway in poultry skeletal muscle physiology and meat quality: an opinion paper

**DOI:** 10.3389/fphys.2023.1228318

**Published:** 2023-07-05

**Authors:** Jiahui Xu, Sandra G. Velleman

**Affiliations:** Department of Animal Sciences, The Ohio State University, Wooster, OH, United States

**Keywords:** meat quality, mTOR, muscle growth, poultry, satellite cells, skeletal muscle

Skeletal muscle is the major component of meat, and is primarily composed of muscle fibers bounded by multiple connective tissue layers (Velleman and McFarland, 2014). These connective tissue layers function as an essential support and functional system, incorporating components like blood vessels and extracellular matrix macromolecules. Consequently, muscle development and growth, morphological structure, and biochemistry are crucial aspects in the determination of meat yield and quality. The modern poultry industry has had one area of focus on selection for enhanced growth performance, specifically emphasizing increased body weight and skeletal muscle yield (Havenstein et al., 2007; Collins et al., 2014). Structural abnormalities, such as diminished connective tissue spacing and reduced capillary density resulting from excessive muscle fiber hypertrophy, have been observed in the breast muscle of modern rapid-growing poultry lines (Velleman et al., 2003; Joiner et al., 2014). The loss of connective tissue spacing and presence of oversized myofibers result in direct contact between muscle fibers, and this condition is correlated with a greater occurrence of muscle fiber degeneration (Wilson et al., 1990; Velleman et al., 2003). Furthermore, insufficient capillary supply in the breast muscle could limit the removal of anaerobic respiration byproducts, such as lactic acid. The residual lactic acid in the breast muscle can lead to a decrease in pH, potentially exacerbating muscle degeneration. In addition to the structural flaws, the breast muscle of modern fast-growing poultry breeds exhibits conditions such as Wooden Breast (Sihvo et al., 2014) and White Stripping (Soglia et al., 2018), which adversely affect the quality of the breast meat. Muscle growth and structure are primarily determined by muscle cell biology and biochemistry, which are influenced by signal transduction pathways. One of the key players involved in muscle function is the mechanistic target of rapamycin (mTOR) pathway, which is critical in regulating muscle hypertrophic growth and mass accretion in poultry (Vignale et al., 2015; Ma et al., 2018). This opinion paper will discuss how the mTOR pathway modulates skeletal muscle growth, structure, and biochemistry, and ultimately can affect poultry meat yield and quality.

Muscle fiber number is fixed by the time of hatch (Smith, 1963). Post-hatch muscle grows through the hypertrophy of existing muscle fibers. Accumulation of intracellular protein in existing muscle fibers is the most likely mechanism for post-hatch muscle hypertrophic growth. With regard to the molecular mechanisms, mTOR is a key regulator controlling muscle size and mass accretion in mammals and poultry (Bodine et al., 2001; Vignale et al., 2015). It has been broadly hypothesized that mTOR promotes myofiber hypertrophy by stimulating protein synthesis (Wang and Proud, 2006; Wang et al., 2015; You et al., 2019). A schematic illustration of possible mechanisms of mTOR pathway in skeletal muscle function is presented in Figure 1. Using mammalian models, the mTOR protein kinase has been found to function in two distinct multiprotein complexes: mTOR complex 1 (mTORC1) and mTOR complex 2 (mTORC2) (Hara et al., 2002; Sarbassov et al., 2004). As an intracellular nutrient sensor (Tesseraud et al., 2006; Vignale et al., 2015), mTORC1 has been found to promote protein synthesis with the stimulation of intracellular nutrients including amino acids (Kop-Bozbay and Ocak, 2019), vitamins (Vignale et al., 2015), fatty acids (Yoon et al., 2011), and glucose (Patel et al., 2001) in birds and mammals. In addition to nutrients, extracellular growth factors also stimulate the activation of mTORC1 via specific transmembrane growth factor receptors (Rommel et al., 2001). Both nutrients and growth factors activate mTORC1 via the phosphoinositide 3 kinase (PI3K)/protein kinase B (Akt) signaling. For mTORC2, it can also be activated by nutrients (Tato et al., 2011) and growth factors (García-Martínez and Alessi, 2008) through the PI3K/Akt pathway in mammalian cells. Activated mTORC2 indirectly activates mTORC1 through Akt (Sarbassov et al., 2005; Moschella et al., 2013). Downstream mTOR effectors for protein synthesis are p70 S6 kinase (S6K) (Brown et al., 1995; Ohanna et al., 2005; Vignale et al., 2015) and eukaryotic initiation factor 4E binding protein 1 (4EBP1) (Hara et al., 1998; Wang et al., 2015). As the amount of intracellular protein directly determines the size of myofibers, both mTOR/S6K and mTOR/4EBP1 signaling plays an essential role in regulating the hypertrophic growth of poultry skeletal muscle (Zhang et al., 2014; Wang et al., 2015). Notably, using human models, Cuthbertson et al. (2006) reported that it is the myofibrillar proteins and not the sarcoplasmic proteins which promote hypertrophic growth of muscle fibers. Abou Sawan et al. (2018) also showed that mTORC1 increased muscle myofibrillar protein synthesis but not mitochondrial protein synthesis via the S6K and 4EBP1 in human muscle fibers. In avian species, the mTOR pathway may also promote muscle fiber hypertrophy and muscle mass accretion by upregulating myofibrillar proteins synthesis in an S6K- and 4EBP1-dependent manner. Future studies will be needed to test this hypothesis.

**FIGURE 1:**
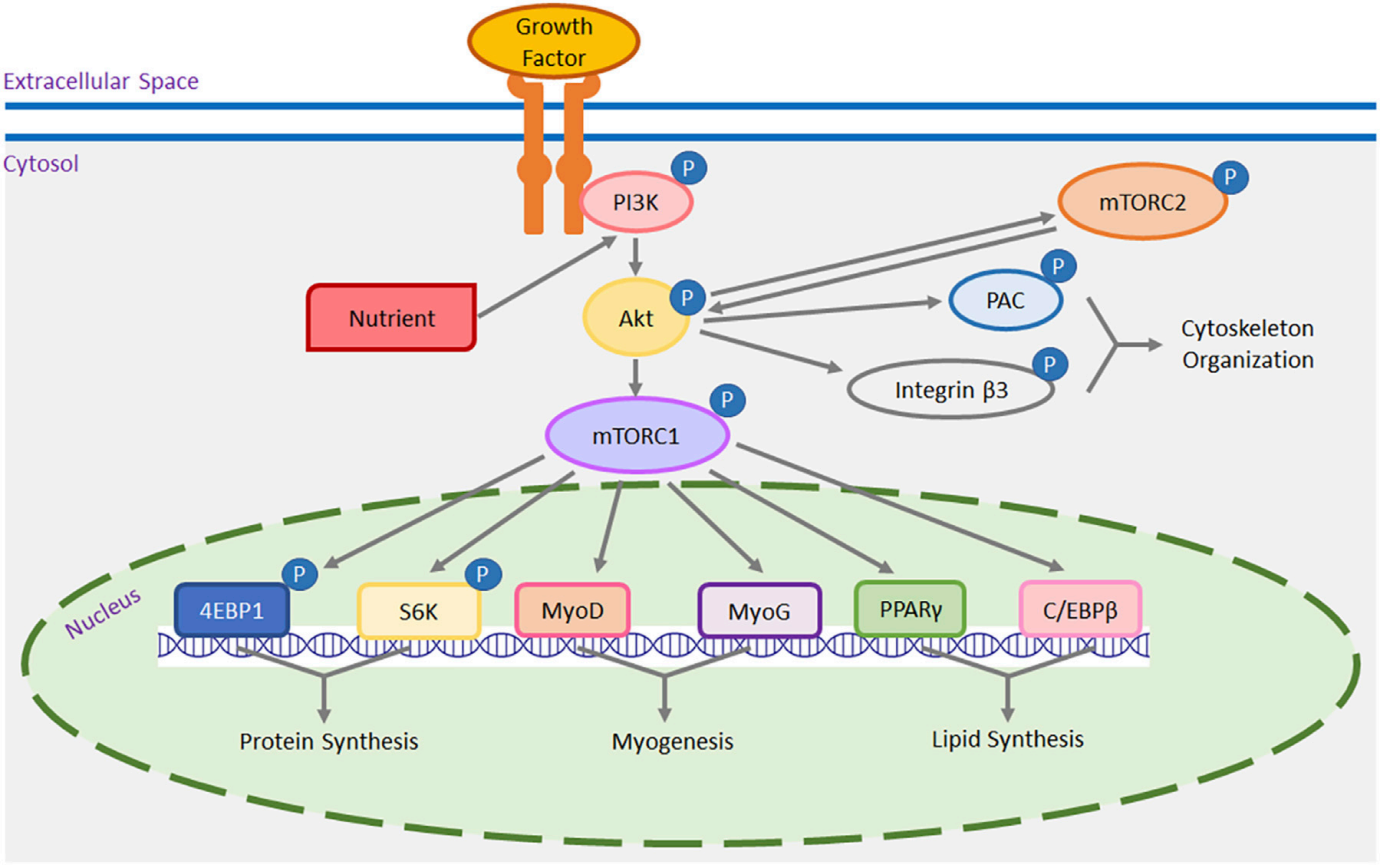
A schematic representation of the mTOR pathway in muscle cells. Both growth factors, through their receptors, and intracellular nutrients trigger the activation (or phosphorylation) of phosphoinositide 3 kinase (PI3K). Once activated, PI3K in turn activates protein kinase B (Akt) and mTOR complex 1 (mTORC1). Nutrients and growth factors, via the PI3K/Akt pathway, can also stimulate mTOR complex 2 (mTORC2), which, once activated, can further stimulate mTORC1 via Akt. Moreover, activated mTORC2 phosphorylates cytoplasmic p21-activated kinase (PAK) and integrin β3 via Akt, contributing to cytoskeleton organization and migration. Downstream targets of mTORC1 include but are not limited to p70 S6 kinase (S6K), eukaryotic initiation factor 4E binding protein 1 (4EBP1), myoblast determination factor 1 (MyoD), myogenin (MyoG), peroxisome proliferator-activated receptor-gamma (PPARγ), and CCAAT/enhancer-binding protein-beta (C/EBPβ). Both S6K and 4EBP1 are implicated in the initiation of gene expression for protein synthesis. MyoD and MyoG are myogenic transcriptional regulatory factors promoting myogenesis, while PPARγ and C/EBPβ are adipogenic factors that stimulate the transcription of genes involved in lipid synthesis.

At the periphery of each muscle fiber, there exists a specific population of muscle stem cells known as satellite cells (Mauro, 1961). Satellite cells act as the exclusive cell reservoir for post-hatch muscle hypertrophy, and this occurs through satellite cell proliferation, differentiation, and donation of cell nuclei to existing muscle fibers (Moss and Leblond, 1971; Cardiasis and Cooper, 1975). In poultry, satellite cell mitotic activity peaks during the first week after hatch (Mozdziak et al., 1994; Halevy et al., 2000), after which it gradually diminishes, eventually reaching a mitotically quiescence state in mature muscle (Schultz and Lipton, 1982). With damage to muscle fibers (Bischoff, 1975; Snow, 1977), the mitotically inactive satellite cells re-enter the cell cycle and repair the damaged muscle fibers. Numerous studies have suggested that mTORC1 promotes satellite cell myogenesis by inducing the expression of myogenic transcriptional factors such as myoblast determination factor 1 (MyoD) and myogenin (MyoG) (Han et al., 2008; Vignale et al., 2015; Xu et al., 2022a; Xu and Velleman, 2023) (Figure 1). In chicken breast muscle, impaired proliferation and differentiation with decreased expression of *mTOR, MyoD,* and *MyoG* were observed in the breast muscle satellite cells of a current faster-growing broiler chicken line compared to two historical chicken lines from 1990s (Xu and Velleman, 2023). Insufficient myogenesis by satellite cells may result in a higher incidence of myofiber degenerative and fibrotic myopathies like Wooden Breast, as satellite cells with impaired regeneration potential are unable to fully restore the necrotic myofibers to their original size (Velleman and Clark, 2015; Clark and Velleman, 2016; Velleman et al., 2018). In contrast, Wooden Breast has not been observed in modern faster-growing turkeys. This difference can be partially explained by increased satellite cell myogenesis facilitated by an enhanced mTOR/S6K pathway in turkeys (Xu et al., 2022a). The other complex, mTORC2, promotes mouse satellite cell myogenesis, primarily through the activation of Akt/mTORC1 signaling (Matheny Jr et al., 2012) (Figure 1). In addition, mTORC2-triggered Akt activation influences actin polymerization, which in turn affects cytoskeleton organization and cell migration through its downstream effectors including p21-activated kinase (PAK) (Zhou et al., 2003) and integrin β3 (Kirk et al., 2000) in mammals (Figure 1). Satellite cell alignment, a prerequisite for their fusion to form multinucleated myotubes, requires migration (Chazaud et al., 1998). Taken together, the mTOR pathway plays a multifaceted role in muscle biology; it not only directly regulates protein synthesis in muscle fibers but also modulates muscle hypertrophy and regeneration potential of damaged fibers by controlling the myogenic or regenerative potential and migration of satellite cells.

In addition to regulating muscle growth and regeneration, the mTOR pathway also governs the possible adipogenesis of muscle satellite cells. This is accomplished by regulating the expression of adipogenic regulatory factors like peroxisome proliferator-activated receptor-gamma (PPARγ) (Kim and Chen, 2004) and CCAAT/enhancer-binding protein-beta (C/EBPβ) (Kim et al., 2014) (Figure 1). As multipotential stem cells, satellite cells can spontaneously transdifferentiate to an adipocyte-like lineage and synthesize lipid content with appropriate extrinsic stimuli (Asakura et al., 2001; Shefer et al., 2004). As shown by Xu et al. (2021; 2022a), heat stress significantly increased the activity of the mTOR/S6K pathway, which is accompanied by increased lipid synthesis in turkey breast muscle satellite cells. Furthermore, knocking down the expression of *mTOR* significantly decreased lipid accumulation and suppressed the expression of both *PPARγ* and *C/EBPβ* in turkey satellite cells (Xu et al., 2022b). In in vivo studies, the increased intracellular lipid content has been associated with the increased intramuscular fat deposition in chicken breast muscle (Piestun et al., 2017; Patael et al., 2019), potentially influencing protein-to-fat ratio in poultry breast muscle. The increase in intramuscular fat depots may also be associated with fat-associated myopathies like White Striping.

Considering the crucial role of the mTOR pathway in skeletal muscle growth, structure, and physiology, numerous extrinsic factors have been investigated for their potential effects on mTOR activity. Nutrients such as phosphatidic acid (Yoon et al., 2011), vitamin D (Vignale et al., 2015) and leucine (Kop-Bozbay and Ocak, 2019) and specific growth factors like epidermal growth factor (EGF) (Cao et al., 2009) and insulin-like growth factor-1 (IGF-1) (Rommel et al., 2001) are well-known activators of the mTOR pathway in birds and mammals, which may in turn stimulates muscle protein synthesis. The mTOR pathway is also significantly influenced by various cellular stressors. For example, the mTOR pathway can sense and respond to thermal stress (Xu et al., 2022a) and oxygen stress (Chaillou and Lanner, 2016), subsequently adjusting protein synthesis in skeletal muscle. Nonetheless, the regulation of the mTOR pathway is tissue- and species-specific in poultry muscle, relying on a delicate balance of various factors. Different timing, intensity, or duration of these stimuli can also result in distinct cellular responses. Taking temperature effect as an example, Xu et al. (2022a) reported that cold stress (5°C colder than the control) inhibited the activity of the mTOR/S6K pathway in breast muscle satellite cells of one-week-old turkeys. However, an increase in mTOR activity was observed when newly hatched chickens were constantly challenged with chronic cold stress (5.3°–12.3°C colder than the control) during the first week after hatch in the chicken leg muscle (Nguyen et al., 2015). Comprehending how the extrinsic factors are involved in the regulation of the mTOR pathway is critical in optimizing poultry skeletal muscle growth and structure.

The mTOR pathway is undeniably critical in poultry skeletal muscle growth and physiology by stimulating myofiber protein accumulation (Wang and Proud, 2006) and regulating satellite cell myogenesis and adipogenesis (Xu et al., 2022a; b). These mTOR functions may have a direct impact on poultry meat quality. Gaining a deeper understanding of the mTOR pathway and its regulatory mechanisms will enable the poultry industry to develop strategies for optimizing poultry muscle growth and enhancing meat quality. For example, providing feed with higher vitamin D (Vignale et al., 2015), arginine (Yu et al., 2018), leucine (Kop-Bozbay and Ocak, 2019) might achieve the nutritional stimuli necessary for mTOR activity in poultry skeletal muscle. Introducing a heat stress with appropriate intensity and duration, particularly during the first week after hatch when satellite cells exhibit peak mitotic activity and temperature sensitivity (Mozdziak et al., 1994; Halevy et al., 2001), will significantly increase the activity of the mTOR pathway (Xu et al., 2022a), which in turn, will stimulate satellite cell myogenesis and protein synthesis, resulting in increased muscle mass accretion and preventing myofiber necrotic and fibrotic myopathies like Wooden Breast. Nevertheless, as indicated by Ma et al. (2018), it is vital to avoid chronic high-intensity heat stress to mitigate negative effects on mTOR activity. Furthermore, the elevation in mTOR activity induced by heat stress at an early age also promotes fat accumulation, particularly in the breast muscle satellite cells of rapid-growing poultry (Xu et al., 2022b). Increased intramuscular fat deposition could be associated with fat-associated myopathies like White Striping, impacting the quality of breast meat. As poultry breast muscle is a favored consumer source of high-protein and low-fat meat, fluctuating between heat and cold stress in the first week post-hatch could potentially augment mTOR-mediated protein synthesis, while inhibiting mTOR-driven fat production. Continued research is necessary to discover the appropriate strategies of controlling the mTOR pathway in response to various stimuli, ultimately improving poultry skeletal muscle growth while producing a high-quality meat product.

## References

[B1] Abou Sawan S. Van Vliet S. Parel J. T. Beals J. W. Mazzulla M. West D. W. (2018). Translocation and protein complex co‐localization of mTOR is associated with postprandial myofibrillar protein synthesis at rest and after endurance exercise. Physiol. Rep. 6, e13628. 10.14814/phy2.13628 29512299 PMC5840389

[B2] Asakura A. Komaki M. Rudnicki M. (2001). Muscle satellite cells are multipotential stem cells that exhibit myogenic, osteogenic, and adipogenic differentiation. Differentiation 68, 245–253. 10.1046/j.1432-0436.2001.680412.x 11776477

[B3] Bischoff R. (1975). Regeneration of single skeletal muscle fibers *in vitro* . Anat. Rec. 182, 215–235. 10.1002/ar.1091820207 168794

[B4] Bodine S. C. Stitt T. N. Gonzalez M. Kline W. O. Stover G. L. Bauerlein R. (2001). Akt/mTOR pathway is a crucial regulator of skeletal muscle hypertrophy and can prevent muscle atrophy *in vivo* . Nat. Cell Biol. 3, 1014–1019. 10.1038/ncb1101-1014 11715023

[B5] Brown E. J. Beal P. A. Keith C. T. Chen J. Bum Shin T. Schreiber S. L. (1995). Control of p70 s6 kinase by kinase activity of FRAP *in vivo* . Nature 377, 441–446. 10.1038/377441a0 7566123

[B6] Cao C. Huang X. Han Y. Wan Y. Birnbaumer L. Feng G. S. (2009). Galpha(i1) and Galpha(i3) are required for epidermal growth factor-mediated activation of the Akt-mTORC1 pathway. Sci. Signal. 2, ra17. 10.1126/scisignal.2000118 19401591 PMC4138699

[B7] Cardiasis A. Cooper G. (1975). An analysis of nuclear numbers in individual muscle fibers during differentiation and growth: A satellite cell-muscle fiber growth unit. J. Exp. Zool. 191, 347–358. 10.1002/jez.1401910305 1127400

[B8] Chaillou T. Lanner J. T. (2016). Regulation of myogenesis and skeletal muscle regeneration: Effects of oxygen levels on satellite cell activity. FASEB J. 30, 3929–3941. 10.1096/fj.201600757R 27601440

[B9] Chazaud B. Christov C. Gherardi R. K. Barlovatz-Meimon G. (1998). *In vitro* evaluation of human muscle satellite cell migration prior to fusion into myotubes. J. Muscle Res. Cell. Motil. 19, 931–936. 10.1023/a:1005451725719 10047992

[B10] Clark D. Velleman S. (2016). Spatial influence on breast muscle morphological structure, myofiber size, and gene expression associated with the wooden breast myopathy in broilers. Poult. Sci. 95, 2930–2945. 10.3382/ps/pew243 27444449

[B11] Collins K. Kiepper B. Ritz C. Mclendon B. Wilson J. (2014). Growth, livability, feed consumption, and carcass composition of the Athens Canadian Random Bred 1955 meat-type chicken versus the 2012 high-yielding Cobb 500 broiler. Poult. Sci. 93, 2953–2962. 10.3382/ps.2014-04224 25352681

[B12] Cuthbertson D. J. Babraj J. Smith K. Wilkes E. Fedele M. J. Esser K. (2006). Anabolic signaling and protein synthesis in human skeletal muscle after dynamic shortening or lengthening exercise. Am. J. Physiol. 290, E731–E738. 10.1152/ajpendo.00415.2005 16263770

[B13] García-Martínez J. M. Alessi D. R. (2008). mTOR complex 2 (mTORC2) controls hydrophobic motif phosphorylation and activation of serum-and glucocorticoid-induced protein kinase 1 (SGK1). Biochem. J. 416, 375–385. 10.1042/BJ20081668 18925875

[B15] Halevy O. Geyra A. Barak M. Uni Z. Sklan D. (2000). Early posthatch starvation decreases satellite cell proliferation and skeletal muscle growth in chicks. J. Nutr. 130, 858–864. 10.1093/jn/130.4.858 10736342

[B16] Halevy O. Krispin A. Leshem Y. Mcmurtry J. P. Yahav S. (2001). Early-age heat exposure affects skeletal muscle satellite cell proliferation and differentiation in chicks. Am. J. Physiol. Regul. Integr. Comp. Physiol. 281, R302–R309. 10.1152/ajpregu.2001.281.1.R302 11404306

[B17] Han B. Tong J. Zhu M. J. Ma C. Du M. (2008). Insulin‐like growth factor‐1 (IGF‐1) and leucine activate pig myogenic satellite cells through mammalian target of rapamycin (mTOR) pathway. Mol. Reprod. Dev. 75, 810–817. 10.1002/mrd.20832 18033679

[B18] Hara K. Maruki Y. Long X. Yoshino K.-I. Oshiro N. Hidayat S. (2002). Raptor, a binding partner of target of rapamycin (TOR), mediates TOR action. Cell 110, 177–189. 10.1016/s0092-8674(02)00833-4 12150926

[B19] Hara K. Yonezawa K. Weng Q. P. Kozlowski M. T. Belham C. Avruch J. (1998). Amino acid sufficiency and mTOR regulate p70 S6 kinase and eIF-4E BP1 through a common effector mechanism. J. Biol. Chem. 273, 14484–14494. 10.1074/jbc.273.23.14484 9603962

[B20] Havenstein G. Ferket P. Grimes J. Qureshi M. Nestor K. (2007). Comparison of the performance of 1966-versus 2003-type turkeys when fed representative 1966 and 2003 Turkey diets: Growth rate, livability, and feed conversion. Poult. Sci. 86, 232–240. 10.1093/ps/86.2.232 17234835

[B21] Joiner K. S. Hamlin G. A. Lien A. R. Bilgili S. F. (2014). Evaluation of capillary and myofiber density in the pectoralis major muscles of rapidly growing, high-yield broiler chickens during increased heat stress. Avian Dis. 58, 377–382. 10.1637/10733-112513-Reg.1 25518431

[B22] Kim J. E. Chen J. (2004). Regulation of peroxisome proliferator–activated receptor-γ activity by mammalian target of rapamycin and amino acids in adipogenesis. Diabetes 53, 2748–2756. 10.2337/diabetes.53.11.2748 15504954

[B23] Kim J. H. Kim S. H. Song S. Y. Kim W. S. Song S. U. Yi T. (2014). Hypoxia induces adipocyte differentiation of adipose‐derived stem cells by triggering reactive oxygen species generation. Cell Biol. Int. 38, 32–40. 10.1002/cbin.10170 23956071

[B24] Kirk R. I. Sanderson M. R. Lerea K. M. (2000). Threonine phosphorylation of the β3 integrin cytoplasmic tail, at a site recognized by PDK1 and Akt/PKB *in vitro*, regulates Shc binding. J. Biol. Chem. 275, 30901–30906. 10.1074/jbc.M001908200 10896934

[B25] Kop-Bozbay C. Ocak N. (2019). In ovo injection of branched‐chain amino acids: Embryonic development, hatchability and hatching quality of Turkey poults. J. Anim. Physiol. Anim. Nutr. 103, 1135–1142. 10.1111/jpn.13111 31050076

[B26] Ma B. He X. Lu Z. Zhang L. Li J. Jiang Y. (2018). Chronic heat stress affects muscle hypertrophy, muscle protein synthesis and uptake of amino acid in broilers via insulin like growth factor-mammalian target of rapamycin signal pathway. Poult. Sci. 97, 4150–4158. 10.3382/ps/pey291 29982693

[B27] Matheny R. W. Jr Lynch C. M. Leandry L. A. (2012). Enhanced Akt phosphorylation and myogenic differentiation in PI3K p110β-deficient myoblasts is mediated by PI3K p110α and mTORC2. Growth factors. 30, 367–384. 10.3109/08977194.2012.734507 23137199

[B28] Mauro A. (1961). Satellite cell of skeletal muscle fibers. J. Cell Biol. 9, 493–495. 10.1083/jcb.9.2.493 PMC2225012 13768451

[B29] Moschella P. C. Mckillop J. Pleasant D. L. Harston R. K. Balasubramanian S. Kuppuswamy D. (2013). mTOR complex 2 mediates Akt phosphorylation that requires PKCε in adult cardiac muscle cells. Cell. Signal. 25, 1904–1912. 10.1016/j.cellsig.2013.05.001 23673367 PMC3704180

[B30] Moss F. Leblond C. (1971). Satellite cells as the source of nuclei in muscles of growing rats. Anat. Rec. 170, 421–435. 10.1002/ar.1091700405 5118594

[B31] Mozdziak P. E. Schultz E. Cassens R. G. (1994). Satellite cell mitotic activity in posthatch Turkey skeletal muscle growth. Poult. Sci. 73, 547–555. 10.3382/ps.0730547 8202434

[B32] Nguyen P. Greene E. Ishola P. Huff G. Donoghue A. Bottje W. (2015). Chronic mild cold conditioning modulates the expression of hypothalamic neuropeptide and intermediary metabolic-related genes and improves growth performances in young chicks. PLoS One 10, e0142319. 10.1371/journal.pone.0142319 26569484 PMC4646505

[B33] Ohanna M. Sobering A. K. Lapointe T. Lorenzo L. Praud C. Petroulakis E. (2005). Atrophy of S6K1−/− skeletal muscle cells reveals distinct mTOR effectors for cell cycle and size control. Nat. Cell Biol. 7, 286–294. 10.1038/ncb1231 15723049

[B34] Patael T. Piestun Y. Soffer A. Mordechay S. Yahav S. Velleman S. G. (2019). Early posthatch thermal stress causes long-term adverse effects on pectoralis muscle development in broilers. Poult. Sci. 98, 3268–3277. 10.3382/ps/pez123 31041445

[B35] Patel J. Wang X. Proud C. G. (2001). Glucose exerts a permissive effect on the regulation of the initiation factor 4E binding protein 4E-BP1. Biochem. J. 358, 497–503. 10.1042/0264-6021:3580497 11513750 PMC1222084

[B36] Piestun Y. Patael T. Yahav S. Velleman S. G. Halevy O. (2017). Early posthatch thermal stress affects breast muscle development and satellite cell growth and characteristics in broilers. Poult. Sci. 96, 2877–2888. 10.3382/ps/pex065 28444312

[B37] Rommel C. Bodine S. C. Clarke B. A. Rossman R. Nunez L. Stitt T. N. (2001). Mediation of IGF-1-induced skeletal myotube hypertrophy by PI(3)K/Akt/mTOR and PI(3)K/Akt/GSK3 pathways. Nat. Cell Biol. 3, 1009–1013. 10.1038/ncb1101-1009 11715022

[B38] Sarbassov D. D. Ali S. M. Kim D. H. Guertin D. A. Latek R. R. Erdjument-Bromage H. (2004). Rictor, a novel binding partner of mTOR, defines a rapamycin-insensitive and raptor-independent pathway that regulates the cytoskeleton. Curr. Biol. 14, 1296–1302. 10.1016/j.cub.2004.06.054 15268862

[B39] Sarbassov D. D. Guertin D. A. Ali S. M. Sabatini D. M. (2005). Phosphorylation and regulation of Akt/PKB by the rictor-mTOR complex. Science 307, 1098–1101. 10.1126/science.1106148 15718470

[B40] Schultz E. Lipton B. H. (1982). Skeletal muscle satellite cells: Changes in proliferation potential as a function of age. Mech. Ageing Dev. 20, 377–383. 10.1016/0047-6374(82)90105-1 7166986

[B41] Shefer G. Wleklinski-Lee M. Yablonka-Reuveni Z. (2004). Skeletal muscle satellite cells can spontaneously enter an alternative mesenchymal pathway. J. Cell Sci. 117, 5393–5404. 10.1242/jcs.01419 15466890

[B42] Sihvo H. K. Immonen K. Puolanne E. (2014). Myodegeneration with fibrosis and regeneration in the pectoralis major muscle of broilers. Vet. Pathol. 51, 619–623. 10.1177/0300985813497488 23892375

[B43] Smith J. H. (1963). Relation of body size to muscle cell size and number in the chicken. Poult. Sci. 42, 283–290. 10.3382/ps.0420283

[B44] Snow M. H. (1977). Myogenic cell formation in regenerating rat skeletal muscle injured by mincing. II. An autoradiographic study. Anat. Rec. 188, 201–217. 10.1002/ar.1091880206 869238

[B45] Soglia F. Baldi G. Laghi L. Mudalal S. Cavani C. Petracci M. (2018). Effect of white striping on Turkey breast meat quality. Animal 12, 2198–2204. 10.1017/S1751731117003469 29306347

[B46] Tato I. Bartrons R. Ventura F. Rosa J. L. (2011). Amino acids activate mammalian target of rapamycin complex 2 (mTORC2) via PI3K/Akt signaling. J. Biol. Chem. 286, 6128–6142. 10.1074/jbc.M110.166991 21131356 PMC3057817

[B47] Tesseraud S. Abbas M. Duchene S. Bigot K. Vaudin P. Dupont J. (2006). Mechanisms involved in the nutritional regulation of mRNA translation: Features of the avian model. Nutr. Res. Rev. 19, 104–116. 10.1079/NRR2006120 19079879

[B48] Velleman S. G. Anderson J. W. Coy C. S. Nestor K. E. (2003). Effect of selection for growth rate on muscle damage during Turkey breast muscle development. Poult. Sci. 82, 1069–1074. 10.1093/ps/82.7.1069 12872961

[B49] Velleman S. G. Clark D. L. (2015). Histopathologic and myogenic gene expression changes associated with wooden breast in broiler breast muscles. Avian Dis. 59, 410–418. 10.1637/11097-042015-Reg.1 26478160

[B50] Velleman S. G. Clark D. L. Tonniges J. R. (2018). The effect of the wooden breast myopathy on sarcomere structure and organization. Avian Dis. 62, 28–35. 10.1637/11766-110217-Reg.1 29620464

[B51] Velleman S. G. McFarland D. C. (2014). “Skeletal muscle,” in Sturkie's avian physiology: Six edition. Editor Scanes C. G. (New York, NY: Academic Press), 379–402.

[B52] Vignale K. Greene E. S. Caldas J. V. England J. A. Boonsinchai N. Sodsee P. (2015). 25-hydroxycholecalciferol enhances male broiler breast meat yield through the mTOR pathway. J. Nutr. Sci. 145, 855–863. 10.3945/jn.114.207936 25788584

[B53] Wang X. Jia Q. Xiao J. Jiao H. Lin H. (2015). Glucocorticoids retard skeletal muscle development and myoblast protein synthesis through a mechanistic target of rapamycin (mTOR)-signaling pathway in broilers (Gallus gallus domesticus). Stress 18, 686–698. 10.3109/10253890.2015.1083551 26371871

[B54] Wang X. Proud C. G. (2006). The mTOR pathway in the control of protein synthesis. Physiology 21, 362–369. 10.1152/physiol.00024.2006 16990457

[B55] Wilson B. W. Nieberg P. S. Buhr R. J. Kelly B. J. Shultz F. T. (1990). Turkey muscle growth and focal myopathy. Poult. Sci. 69, 1553–1562. 10.3382/ps.0691553 2247418

[B56] Xu J. Strasburg G. M. Reed K. M. Velleman S. G. (2021). Effect of temperature and selection for growth on intracellular lipid accumulation and adipogenic gene expression in Turkey pectoralis major muscle satellite cells. Front. Physiol. 12, 667814. 10.3389/fphys.2021.667814 34140894 PMC8204085

[B57] Xu J. Strasburg G. M. Reed K. M. Velleman S. G. (2022a). Thermal stress affects proliferation and differentiation of Turkey satellite cells through the mTOR/S6K pathway in a growth-dependent manner. PLoS One 17, e0262576. 10.1371/journal.pone.0262576 35025965 PMC8758067

[B58] Xu J. Strasburg G. M. Reed K. M. Velleman S. G. (2022b). Thermal stress and selection for growth affect myogenic satellite cell lipid accumulation and adipogenic gene expression through mechanistic target of rapamycin pathway. J. Anim. Sci. 100, skac001. 10.1093/jas/skac001 35908789 PMC9339274

[B59] Xu J. Velleman S. G. (2023). Effects of thermal stress and mechanistic target of rapamycin and wingless-type mouse mammary tumor virus integration site family pathways on the proliferation and differentiation of satellite cells derived from the breast muscle of different chicken lines. Poult. Sci. 102, 102608. 10.1016/j.psj.2023.102608 36948037 PMC10033751

[B60] Yoon M. S. Sun Y. Arauz E. Jiang Y. Chen J. (2011). Phosphatidic acid activates mammalian target of rapamycin complex 1 (mTORC1) kinase by displacing FK506 binding protein 38 (FKBP38) and exerting an allosteric effect. J. Biol. Chem. 286, 29568–29574. 10.1074/jbc.M111.262816 21737445 PMC3190997

[B61] You J. S. Mcnally R. M. Jacobs B. L. Privett R. E. Gundermann D. M. Lin K.-H. (2019). The role of raptor in the mechanical load‐induced regulation of mTOR signaling, protein synthesis, and skeletal muscle hypertrophy. FASEB J. 33, 4021–4034. 10.1096/fj.201801653RR 30509128 PMC6404572

[B62] Yu L. Gao T. Zhao M. Lv P. Zhang L. Li J. (2018). Effects of in ovo feeding of l-arginine on breast muscle growth and protein deposition in post-hatch broilers. Animal 12, 2256–2263. 10.1017/S1751731118000241 29478426

[B63] Zhang R. P. Liu H. H. Li Q. Q. Wang Y. Liu J. Y. Hu J.-W. (2014). Gene expression patterns, and protein metabolic and histological analyses for muscle development in Peking duck. Poult. Sci. 93, 3104–3111. 10.3382/ps.2014-04145 25306455

[B64] Zhou G. L. Zhuo Y. King C. C. Fryer B. H. Bokoch G. M. Field J. (2003). Akt phosphorylation of serine 21 on Pak1 modulates Nck binding and cell migration. Mol. Cell. Biol. 23, 8058–8069. 10.1128/MCB.23.22.8058-8069.2003 14585966 PMC262366

